# Reflexive expectations in innovation financing: An analysis of venture capital as a mode of valuation

**DOI:** 10.1177/03063127221118372

**Published:** 2022-08-15

**Authors:** Kean Birch

**Affiliations:** York University, Toronto, ON, Canada

**Keywords:** reflexive expectations, innovation financing, venture capital, life sciences, technoscientific capitalism

## Abstract

Social studies of expectations are premised on the notion that the future is brought into the present, and thereby expectations about the future come to shape our actions, decisions, and practices in ways that performatively bring about the imagined future. In this article, I examine how social actors themselves understand, construct, and deploy future expectations in innovation financing, focusing specifically on the venture capital industry financing of the life sciences sector. I do so to analyse how these reflexive efforts configure the valuation and investment decisions of these social actors and others. I build on analytical perspectives in STS and adjacent fields such as organization studies and economic sociology that analyse the role of expectations – manifested as stories, narratives, and accounts – in social action. To do so, I unpack how reflexivity comes to configure valuation and investment decisions, and the goals (e.g. exits) they rationalize.

## Introduction

Where do unicorns come from? This is surely a simple question with a simple answer: Unicorns are mythical creatures that do not exist except in stories and tales of adventure and daring-do. Yet, there are other kinds of unicorns that *do* exist, and also exist in stories and tales of adventure and daring-do, laced with moral overtones of changing the world for the better – one technology at a time. In these latter stories, a unicorn is understood as an entrepreneurial and highly valued startup, usually in a high-tech sector, which is both worth over US$1 billion and still a private firm (Geiger, 2020; Hogarth, 2017; Kenney & Zysman, 2019). Today, there are close to 1000 unicorns in the world, most being based in the USA or China, although a smattering are to be found in the UK, India, Germany, and South Korea. Unicorns come in many shapes and sizes, but nearly half are in the fintech, internet, e-commerce, and artificial intelligence fields, although ByteDance – parent firm of TikTok – represents a significant proportion of the AI field. Interestingly, although there is a diversity of investors in these unicorns – over 600 in fact – several key venture capital investors crop up again and again, having made multiple investments in a range of unicorns: for example, Sequoia Capital, SoftBank Group, Accel, and Andreessen Horowitz are some key financiers.

*These* unicorns are a new phenomenon, so we can go back to my opening question and ask it again: Where do unicorns come from? Unicorns are the result of a particular mode of valuation characteristic of venture capital. Here, the answer relates to the techno-economic configuration of contemporary, technoscientific capitalism, especially how founders, investors, stockbrokers, analysts, and others understand, construct, and deploy various expectations in the financing process. Venture capital is often associated with uncertainty and risk-taking – rushing into the unknown in the stories of daring-do mentioned above – especially in the minds of policymakers and politicians. It is especially associated with the entrepreneurial, the novel, and the technoscientific (Shapin, 2008), and often represents the fount of high-tech innovation in economies otherwise stagnant and unable to deal with uncertain futures in ways that allow those futures to happen. Venture capital is, in this sense, a useful example of how capitalism and technoscience have become increasingly entangled with one another, making it difficult to distinguish between the two (Birch, 2017b; Birch & Muniesa, 2020). As a mode of valuation, venture capital has centred certain financial logics at the heart of technoscience, such that research and development are increasingly understood as financial processes, representing forms of technology risk or market risk for firms (Fabozzi, 2016; Styhre, 2021).

As a mode of valuation, venture capital entails expectations about the future, usually manifested in the stories, narratives, and accounts – henceforth ‘stories’ – told about and within a particular sector. These stories are part of how social actors both understand and act in the world. And, importantly, this includes their use of stories and a reflexive understanding of their use in investment and valuation judgements. In this article, then, I analyse how innovation financiers, especial venture capitalists, understand, construct, and deploy expectations – manifested as stories – in their investment and valuation decisions. As such, I build on the work of such scholars as Beckert (2013, 2016), Borup et al. (2006), and van Lente (1993) by examining what I define as the ‘reflexive expectations’ that underpin venture capital as a mode of valuation (Tellmann, 2022). My approach is to draw on the existing analytical perspectives in this literature – characterized by an appreciation of the performative nature of such stories – and to draw on emerging research on reflexivity in social studies of finance (e.g. Muniesa & Doganova, 2020; Tellmann, 2020). The empirical information in this article is derived from a long-running project on the implications of the 2007-2008 global financial crisis for innovation financing, specifically in the life sciences sector. Starting in 2011, I began interviewing a range of social actors situated in the venture capital industry, primarily venture financiers of various sorts but also other relevant stakeholders (e.g. trade associations, lawyers, analysts, brokers, etc.). I interviewed 13 UK informants in 2012, 11 Canadian informants in 2016, and 2 EU informants in 2017 – making a total of 25 informants. During the interviews the informants often started talking about the ‘stories’ that startups and financiers construct and deploy about startups, and which serve different roles in the venture investment process.

## Reflexive expectations

The importance of stories in our economies is a longstanding topic within a range of disciplines. Czarniawska (2004), for example, has written extensively about their role in organizational settings. Describing the ‘narrative turn’ in the social sciences, Czarniawska highlights the importance of semiotics in political-economic life; as she points out, social actors actively construct stories and narratives (p. 5). Such stories are not simply subjective, reflecting our own understanding of a situation or desire for a particular outcome, they are intersubjective reflections of negotiations and contestations that play out in contextually specific ways. Actors construct stories deliberately and for specific reasons. Stories are important because they provide meaning, legitimation, and accountability for social activities, decisions, and relations (Palo et al., 2020); that is, they make social action meaningful to people, they legitimate social action, and they hold people to account for their social interactions. All of this has political-economic effects and implications, especially when it comes to the valuation of hi-tech startups.

Palo et al. (2020) argue that even when social actors know that something is ‘unreal’, they still play along and thereby reinforce the political-economic order. Stories play a performative role in making myths real in a techno-economic sense, according to Palo et al.; for example, stories lead businesses to establish flights and itineraries for tourists to visit Santa Claus’s workshop in Lapland. Stories do not have to be about myths, however, they can be more mundane, reflecting narratives about an organization, its purpose, goals, and achievements – all of which are implicated in valuation decisions. For example, Chapple et al. (2021) point out that stories are particularly common in hi-tech sectors, where it is often difficult to identify techno-economic outcomes clearly (see Doganova & Eyquem-Renault, 2009; Muniesa et al., 2017). Here, stories about the future of a technology can play an important role in attracting investment, especially the often necessarily speculative stories told by hi-tech startup founders. Chapple et al. (2021) argue that actors such as financial investors and financial analysts understand that they are being told a story and will reinforce or contest that story in the financing process, often leading to an adjustment or revision of the story. As such, stories are part of the actors’ decision-making, and in a way that can be highly reflexive, which I come back to below.

It is important to stress that this approach to stories treats them very much as constitutive discourses, configuring and reconfiguring market actions and judgements in specific ways. This constitutive framing comes out in a number of other conceptual approaches that emphasize the importance of analysing the role of the future in the present, especially in the more critical literature on emerging technologies from science and technology studies (STS) and economic sociology (see Birch, 2017a; Tutton, 2017). In STS, this literature comes under the umbrella term ‘sociology of technological expectations’ (STE), while in economic sociology it is generally associated with the work of scholars on ‘fictional expectations’ (e.g. Beckert, 2013, 2016). In both literatures, expectations play a central analytical role in the future development, direction, and configuration of our political economies; the former focuses on technoscientific expectations, while the later focuses on political-economic ones.

The STE literature illustrates the longstanding interest in future expectations. Originating in the 1990s with work by van Lente (1993) and van Lente and Rip (1998), STE has made important contributions to understanding the emergence and commercialization of technologies (see Borup et al., 2006). For example, van Lente and Rip (1998, p. 221) argue that expectations embedded in stories, rhetoric, and promises require actors to ‘position themselves with respect to a future technology’. As such, expectations play a key role in bringing actors together in the pursuit of collective goals. Later work provides a range of case studies of the implications of expectations and promises for the development of a range of technologies (e.g. Brown, 2000; Brown & Michael, 2003; Hedgecoe & Martin, 2003; Selin, 2008). A review of the literature by Borup et al. (2006) makes a distinction between the more ‘rational’ and ‘descriptive’ approaches to understanding the future and the more ‘constitutive’ and ‘performative’ approaches. There are a number of aspects to these performative expectations that are worth highlighting. First, techno-economic expectations enroll diverse actors – scientists, investors, politicians, publics – in the pursuit of specific agendas defined by promissory stories/discourses centred on future, and therefore unknown and uncertain, outcomes; as a result, they ameliorate the potential for contestation or conflict over current investments (Borup et al., 2006, p. 289). Second, techno-economic expectations also entail assumptions about what counts as acceptable knowledge claims and who counts as acceptable knowledge makers (Birch, 2017a); that is, who gets to make stories and what stories do they get to make. For example, the oft-derided linear model of innovation – e.g. science → innovation → market – remains embedded in science policy despite longstanding critiques; it reflects a simple story, easy to tell in particular circles (e.g. policy-making), and convenient to tell by particular actors (e.g. universities, investors, entrepreneurs; Mirowski, 2012). Finally, techno-economic expectations are bound up with biophysical materialities (Selin, 2008; Tutton, 2017), entailing interdependencies that lock in agendas, decisions, and actions as they become (largely) irreversible over time.

In economic sociology, Beckert (2013, 2016, 2020) makes the case for considering the central importance of *fictional* expectations – as opposed to rational expectations in mainstream economics – in the economy. With reference to technological innovation, for example, Beckert (2016, p. 179) discusses the role of promises and expectations in the ‘hype cycle’, emphasizing that such (fictional) expectations are ‘collective projections’ rather than stories of heroic, Schumpeterian entrepreneurs. As such, Beckert highlights the importance of connecting fictional expectations to their ‘socially given world’ in order for them to be ‘enacted’ (p. 181). Here, Beckert’s (2016) argument is that fictional expectations – characterized as the stories actors tell – are an important social means to manage the vagaries and unforeseeable outcomes of political-economic uncertainty – defined in the Knightian sense (p. 43). Uncertainty means we cannot assess social action on the basis of some form of calculation, because when we orient towards the future we are dealing with an unknowable and contingent set of conditions and effects. Stories – or fictional expectations – help us to manage this uncertainty by providing more than descriptive narratives; they are constitutive or performative. An example of this is provided by Leins (2018) in his ethnography of financial analysts in a Swiss bank. Leins (2018) shows how analysts, especially the experienced ones, ‘construct a persuasive story’ that is deliberately designed to ‘persuade investors’ and thereby shape the market (pp. 105, 106). They do so by following a particular narrative structure (e.g. start with a hook), deploying plot items (e.g. graphs), and including interesting characters (e.g. CEOs). The content of these stories can be diverse, with Geiger (2020) outlining the important role of adversaries in narratives of technoscientific disruption; for example, in the case of Theranos, considered by Geiger, criticism could be turned against detractors, who were merely sniping at a startup that was challenging incumbents.

Both these bodies of literature on future expectations provide a helpful starting point for an analysis of venture capital. Their analytical arguments can be extended, though, with the consideration of reflexivity, which is emerging in more recent social studies of finance. Reflexivity is the idea that social actors change their actions in response to knowledge claims about the world (Giddens, 1984). Reflexivity is particularly important to consider when it comes to the discussion of expectations, because it implies that expectations are not constitutive or generative as previously theorized, since the stories, narratives and knowledge claims that underpin them can reconfigure the world although not necessarily performatively. That is, social actors change their decisions in response to those expectations, but not to reinforce those expectations. This is particularly relevant when considering venture capital as a mode of valuation, because its valuation practices are defined by an orientation to the future (e.g. present value; Styhre, 2015, 2021; also Fabozzi, 2016; Klonowski, 2018). This has implications not only for the actors, but also for our own understanding of valuation (Tellmann, 2022).

On a theoretical level, Muniesa and Doganova (2020) argue that this emphasis on the future – and its enactment in the present – as the basis for understanding valuation practices can result in a problematic centering of economistic notions of the future in our thinking. As such, they argue that this reinforces the financial analytical lens, rather than challenging it. For example, when valuation is framed as an effect of anticipated future yields, this legitimates the metrics of financial valuation, like return on investment, because it ‘is responsible for producing a certain idea of time’ (Muniesa and Doganova, 2020, p. 108), rather than time producing a certain idea of value. Here, it is important to return to the reflexive construction of expectations, valuations, and futures, or, in Loza’s (2021) terms, how the future is ‘continually (re)articulated, materialized, and embedded in practices and relations’. Social actors understand the future, seek to enact or contest it, and understand it as a construct of their visions, enactments, and contestations. Analysing how they reflexively bring the future ‘into being’ necessitates what Tellmann (2020) theorizes as a ‘durational’ analytic, requiring us to examine the practices, processes, and discourses that stretch from now into the future – and not simply to analyse how *the* future is enacted in *the* present. Stories are an important part of these durational modes, and not just or primarily a form of collective meaning-making that enables certain actors to enrol others in their visions. Stories are ‘knowledge claims’ about the world that transform the world as actors reflexively and knowingly use those stories in an attempt to extend and bind futures to particular objectives.

## Venture capital as a mode of valuation

### A brief introduction to venture capital financing

A common narrative in technoscientific sectors – ranging from life sciences to digital tech – places venture capital (VC) as the financier par excellence, the fount of not only capital for startups but also of expertise, drive, and kudos. This is evident in cultural depictions – for example, HBO’s *Silicon Valley* television series – as well as academic interest in VC as a distinct source of technoscientific financing (e.g. Kenney & Zysman, 2019; Owen & Hopkins, 2016; Pisano, 2006; Shapin, 2008). Indeed, VC plays an inordinately influential role in configuring the financing of technoscience (Balzam & Yuran, 2022; Cooiman, 2021; Hellman, 2022; Sauter, 2020), largely as a mode of valuation configured by specific stories and financial frameworks. VC is a distinctive source of private finance, contrasting not only with public finance from equity or capital markets but also with other forms of private financing that ranges from the 3Fs – or ‘friends, family, and fools’ – through business angels or wealthy individuals to contractual or collaborative partnerships with other, often larger, firms (Birch, 2016). However, VC is not the most important source of financing for technoscience firms; the life sciences sectors, for example, depend more on debt and partnerships to finance their operations (Birch, 2016, 2017b; Fabozzi, 2016; Hopkins, 2012).

At its simplest, VC is the private capital used to invest in private firms. According to the 2021 *Yearbook* produced by the US National Venture Capital Association (NVCA, 2021), American venture investors raised close to US$75 billion in 2020, spread between 339 venture funds. Approximately 11,000 firms received US$164 billion in venture capital in 2020, with some so-called ‘unicorns’ – defined as private firms valued at US$1 billion or more – receiving 31 percent of that capital despite representing a tiny proportion of overall deals. According to CB Insights (2021), these unicorns are predominantly found in the FinTech, internet software, e-commerce, and artificial intelligence sectors, as well as being usually located in the USA or China. As a comparison, Canadian venture investment represented around US$3.2 billion in 2020 spread over 509 deals, representing a fraction of total US venture investments (Canadian Venture Capital Association, 2021); and UK venture investment represented US$15 billion in 2020. As this makes evident, the USA is the epicentre of the venture capital *industry* in the world.

Venture capital is frequently equated with a particular investment pattern, referred to as the ‘power law’ by Mallaby (2022) in his book on the history of VC in Silicon Valley: Returns on VC investments do not follow a normal distribution, but rather very large returns are concentrated in a few investments. Mallaby illustrates this in relation to the investments made by VC firm Horsley Bridge, which invested in ‘7,000 startups between 1985 and 2014’ of which ‘just 5 percent of the capital deployed, generated fully 60 percent of all the Horsley Bridge returns’ (p. 8). This ‘power law’ forms the basis of VC logic: that is, invest in multiple firms but only expect one or two to provide the returns necessary for the success of the fund (Sauter, 2020). When it comes to financing technoscience, some STS scholars argue that this VC logic represents a way of managing uncertainty, largely resulting from the difficulty inherent in valuing new technologies (Shapin, 2008; see also Doganova & Eyquem-Renault, 2009; Doganova & Muniesa, 2015).

This VC logic has led to a more recent focus on scaling up startups quickly to become dominant firms in their market; first as unicorns, then as public corporations (Balzam & Yuran, 2022; Pfotenhauer et al., 2022). Expectations are an important component of this VC logic. As a mode of valuation, VC is configured by the incorporation of future expectations into investment operations, practices, and organizational structures (Beckert, 2013). For example, uncertainty, scaling up, and expectations are reflected in the ‘term sheet’, the document used by VC investors and investees to define, narrate, and perform/constitute their future expectations (Fabozzi, 2016). This term sheet itself is a non-binding agreement that sets out the expectations for a future financial relationship. I unpack this mode of valuation in the following section by outlining how VC funds are formed, how they identify investment options, and how they make investment decisions. Through an analysis of this financing process, I focus on how the social actors involved understand and reflexively use and act upon the future expectations manifested as stories.

### Stories in technoscientific financing

Venture capital investment is represented by a series of distinct funds; for example, in 2020 there were 339 VC funds in the USA. These funds secure capital investment from wealthy individuals and families as well as from financial institutions like pension funds, mutual funds, and the like (Elder-Vass, 2021). Capital investors in VC funds are called *limited partners* (LPs), while the people running the fund are called *general partners* (GPs; Klonowski, 2018). The venture investor Elizabeth Yin provides a useful rundown of the relationship between LPs and GPs in a Twitter thread she wrote, and on which I draw here (Yin, 2021). As Yin points out, most LPs in the USA have to be ‘accredited investors’, meaning that they need to have over US$1 million in assets (outside their principal residences) and an annual salary of over US$200,000 (Fabozzi, 2016). Becoming an LP means committing your capital to the VC fund, but it does not necessarily mean sending that capital to the fund right away; it is a commitment to fund rather than a transfer of funds. As a result, VC funds do not have an enormous amount of capital on hand, which increases their overall rates of return. Yin and others (e.g. Klonowski, 2018) also explain that GPs charge annual management fees – usually 2 percent per year – and expect the fund to last 10 years, which necessarily reduces the funds available to make venture investments to 80 percent of the committed capital – the other 20 percent comprises the management fees. There are other costs too, which means the eventual amount that a venture fund has available often comes to about 75 percent of the LPs’ commitment. As Yin notes, this means that any VC fund has to do well even to break even; that is, invest 75 percent but yield 100 percent of the LPs commitment. The timeframe for achieving these investment returns is usually 3-5 years, and, as one informant noted, this means that over the 10-year fund cycle GPs need quick investment returns in order to recycle capital into new investments to maintain their management fees (VC Investor R).

As a result, VC funds focus on ‘hot’ sectors to make investments; they need to ensure high returns, which are more likely in sectors with lots of investor interest. These expectations are embedded in the general stories that actors tell about innovation financing. At a basic level, these stories provide a collective starting point for investment decisions through the identification of fashionable hi-tech sectors. This is important because VC investors deliberately seek to identify and invest in startups that they think other investors will want to invest in or buy in the future. Otherwise, they might end up with no ‘exits’ (see below), no points at which investors sell their investment and create value, both monetary and societal (Shapin, 2008). Several informants, for example, noted the dilemma of investing in startups where there was no clear expected exit (e.g. Fund of Fund Investor Q; VC Investor R). Klonowski (2018) argues that VC often entails this sort of ‘group mentality’, focusing on ‘darling sectors’ and entailing a collective set of narratives to drive their actions and decisions (p. 162). This is especially important in sectors where there are limited results, financial or otherwise, since the lack of technoscientific development and commercialization limits the capacity to judge past success (Birch, 2017b; Doganova & Muniesa, 2015; Muniesa et al., 2017).

STS scholars like Fortun (2001, 2008) have written about the importance of ‘story stocks’ as a reflection of these techno-economic promises, which is reiterated by the work of Hogarth (2017) and Geiger (2020) on the emergence of ‘unicorns’ and Silicon Valley respectively. As several informants noted, certain hi-tech sectors, such as the life sciences, provide ‘better stories’ than others. For example:

Life science companies are unusual; they tell such good stories. It’s a good pitch to say, ‘I discovered a cure for cancer’, it’s a good story that any investor can relate to. It is not so hard … it’s harder for an IT company to say, ‘I’ve discovered a new, faster way to send packets of data through to big storage systems’ … it doesn’t sell such a good story to a small investor, but there does always seem to be a new crop of investors to pump prime these businesses. (VCT Investor E)

This informant worked at a UK-based venture firm that derived a significant proportion of its investment funds (circa 75 percent) from public funds provided by the UK government. Their main investment strategy was to find startups developing ‘disruptive technologies’ that threatened incumbents, with the goal of selling those startups to the incumbents or their competitors. The ‘good stories’ they talked about shape government investment decisions, with significant UK government funding going to sectors like the life sciences because they are seen as having national significance (Birch, 2016). Similarly, good stories can shape the public market; the same informant noted that many public life science corporations needed to ‘keep that price driving upwards’ with news about future expectations. But there is always a double-edge to these expectations; where they are not met, they can lead to disillusionment with a firm or even sector.

Good stories have a dual character, analytically speaking, being about both past achievements and future expectations (Beckert, 2013; Birch, 2017a). As Beckert (2011) notes, value and valuation are cognitive effects, reflecting collectively generated social conventions or meaning. Good stories are part of this collective process of meaning-making, being repeated in different settings to reinforce the potential value of/in one sector or another. As one informant highlighted:

Everyone likes success, so you only need one or two success stories in a particular space, whether it’s the life sciences or something else, and it attracts attention and it gains momentum. (Venture Debt Investor D).

As a venture debt investor, this informant was slightly unusual; they provided debt financing to life sciences firms, which both founders and other investors found attractive because it did not threaten to dilute equity value. As their quote illustrates, ‘success stories’ both narrate past achievements but can be *used* to stimulate future action. Such stories have a particular temporality and reflexivity to them, illustrating the distinctiveness of VC as a mode of valuation (Muniesa & Doganova, 2020; Tellmann, 2022). In venture capital, past-facing stories legitimate future expectations, especially the potential of sector-specific startups. At the same time, future-facing stories constitute an important element in valuation judgements. While this might be technical calculations of future revenues (e.g. discounted cash flows) – a manifestation of the future in the present through calculations of future yield (Muniesa & Doganova, 2020) – it can also entail strategic choices, like focusing on certain diseases rather than others. For example, a Canadian informant, who had worked in both a VC fund and in a drug development firm, highlighted how research can end up being redirected because of concerns about financial risk: ‘So, we’re leaving off really great diseases, we’re focusing on rare diseases, which has totally pumped up, you know, costs’ (ex-VC Investor N). Here, then, expectations are central to VC as a mode of valuation, but can legitimate an increasingly problematic tendency to ‘destroy economic value’ and ‘drive toward social disruption without social benefit’ (Kenney & Zysman, 2019, p. 5).

### Constructing stories, constructing expectations

Once a VC fund has been formed and received its capital commitment, its general partners (GPs) need to make investments in startups. Most VC investors expect a high failure rate in their investments with the occasional ‘home run’ to make up for the ‘strikeouts’. A frequently cited rule is that 30 percent of investments will be total failures, 30 percent will be marginal failures, and 40 percent will make significant returns (Shapin, 2008, p. 273). While this is framed as a result of market imperatives, these ratios are an effect of the VC mode of valuation itself and not the imperative driving them (Klonowski, 2018). Klonowski argues that VC investors not only overvalue their portfolios, with problematic outcomes, but also dump investments early due to their ‘’spray and pray’ mentality toward investing’ (p. 16). Some investments that need more time to come to fruition end up jettisoned too early. As a result, VC investors construct deals in specific ways, primarily around startups that are expected to generate high and fast revenue growth (also Kenney & Zysman, 2019). Klonowski (2018, p. 127) calls this an ‘accelerated value growth pattern’ that is usually short- to medium-term in outlook, seeks to maximize profits, and to increase cash flow at the expense of investment in things like R&D and innovation.

As a mode of valuation, VC entails that social actors construct or deploy stories that constitute particular expectations, reflecting a financial logic of cost reduction and bumping growth projections immediately before divestment, whether that is through an initial public offering (IPOs) or the more likely trade sale. Stories show up repeatedly in the construction of a startup’s business plan and their pitches to venture and other investors, but here the stories are not narratives of past achievements but rather accounts of fictional expectations (Beckert, 2013, 2016). Notably, these expectations are understood as performative by the actors themselves, illustrating a reflexive underpinning to VC as a mode of valuation. An interesting example is offered in the semi-autobiographical account by Liu (2020) of her time in a ‘tech’ startup, in her book *Abolish Silicon Valley*. She recounts the evolution of the startup in which she was involved and specifically notes that in early stages the ‘pitch decks’ to potential investors ‘were not meant to describe reality; they created reality’ (p. 94). Liu (2020) highlights that the ‘goal was to sell a vision’ (p. 94). Startups are, necessarily, reflexive in their performative ambitions to shape the world through the stories they tell. One informant emphasized the importance for startups of ‘modelling’ their performance ‘through all the investment rounds’ (ex-VC Investor N), constructing a story about their firm’s trajectory ‘from exit to the beginning’ (see next section). How these early stories are constructed and then evolve is an interesting process in itself, and one in which a variety of other actors necessarily become involved.

While a startup’s story might start out as a business plan (Shapin, 2008), it will shift as the startup seeks to become an investible proposition and face challenges to its early narratives and models (Chapple et al., 2021). Unlike the business plan, the investee ‘story’ becomes a collective process for constructing expectations, bringing a wider array of actors into the process, especially financial experts who act on behalf of the startup (Elder-Vass, 2021). In asking one informant how an investee story is constructed, they responded:

It’s different in each occasion. We look at numerous businesses and we think of how achievable they are. You know can we do the deal? But we don’t get paid unless we are successful, so there’s no point us taking on a task if we don’t think we can do it. So we look at what the tools are, what the management team is, have they made money beforehand, can we access stones is a particular angle, is it a particularly sexy area, is there someone else to leverage off who’s made money in a similar area and try and come up with a strategy. (Broker H)

This informant worked at a UK brokerage house where their job was to get startups ready for sale through an IPO or trade sale (see Birch, 2017b). They understood and framed the difference between these exits as the need for a story to appeal to ‘generalist’ or ‘specialist’ audience respectively; the latter would always lead to a higher return, according to this informant’s colleague, because ‘trade buyers trade on superior knowledge’ (Broker I). This required the broker to construct a ‘value inflection point that’s visible to investors’, whoever they are, as this then frames the story they were trying to tell to different audiences.

Here, stories are not to be conflated with fictions or myths (Palo et al., 2020). In fact, startup founders are increasingly expected to be able to understand their ‘market’ by ‘speaking with commercial people, understanding reimbursement dynamics early on, even when you’re at the preclinical level’ (VC Investor R). Another informant, a partner in a major accounting firm (Accountant O), emphasized that startups need to ‘prove your story’. This informant went on to note:

So even early in development, [you have to] understand your product’s contribution and why payers [e.g. patients, hospitals] would value it. So [you need to] solicit input from key stakeholders and key stakeholders include the payers, especially in the US.

As this quote illustrates, expectations are framed by reference to existing markets or market comparators, as these details help to contextualize the investee story. Consequently, startups seeking investment often have to reframe their story reflexively to fit a specific set of financial expectations (e.g. what is their market? What will payees be willing to pay?), as opposed to technoscientific expectations: the same informant noted, for example, that ‘they [i.e. startup founders] had to understand what the strategic and financial investors were looking for’. A less prosaic comment of this reframing was presented by another informant:

We help hone that story, hone that message, call bullshit, give them a cold shower. You know, we’ll get a lot of ventures, you know, fork in the road where they, like they literally could pivot either way, ‘do I go US, do I go for China’. Those are two very different paths for an investor, for everything. We’ll bring in, you know, we’ll do a SWOT session, all hands on deck, and we’ll thrash it out, right. (Incubator P)

This informant worked out of a government-supported incubator, providing seed-stage funding and strategic support for startups. Part of that support was fine-tuning investee pitches. The informant stressed the need for startups to be realistic and specific in constructing their stories, for example by focusing on the ‘total addressable market’ rather than a speculative market of ‘seven bazillion dollars’.

The reason for all of this is that startups need to develop a story that venture investors and brokers can then take to other investors, such as pension funds, mutual funds, etc., or potential buyers in search of an exit. This entailed the creation of prospectuses for ‘roadshows’ which are another important part of the financing process for startups and VC investors as they construct a particular exit option *and* valuation to go with it. This informant highlighted the importance of these materials and events:

I think we did about eight or so drive-on presentations until we thought they’d shape that story into a credible proposition. And that involved initially the life sciences team. But the other part we should just talk about, we got the whole sales team there to make sure that we dumbed down enough …. The vast majority of these investors are generalists, not specialists. So, you know, it needs to be in a story which is accessible to someone who has a very limited knowledge of the sector. (Broker H)

For the informant, the story cannot simply be, ‘[We have] another drug in early-stage development; can we have some money please and you’ll see some returns in 2023 [11 years after the date of the interview].’ These roadshows speak to Elder-Vass’ (2021) argument that the creation of an investor audience – or ‘asset circle’ in his terms – is an important part of the financing process because it increases the ‘supply’ of potential investors (i.e. interested parties). Expanding the investor audience is an important way to increase the valuation of a startup because it generates more potential investors and interest, which pushes up valuations. The conversation between these two informants provides an overview of this process, illustrating the considerable effort, necessary financial connections, and time needed to generate an audience:

Respondent 1: And again, a transaction which was recently done – I won’t put a name to it – to raise sort of £10 to £20 million range, we probably spoke to what, 50 institutions?
Respondent 2: No we went to 90 people.
Respondent 1: Ninety. And it was probably taken by 15.
Respondent 2: We had 35 meetings, something like that, or 30.
Respondent 1: I got 15 people to invest in [Firm X]. We will be sensitive about…
Respondent 2: Yes, that’s right.
Respondent 1: But I’m trying to put a real flavour on that. And in that, we look back, you know if I look back at the quality of that list or the shape of that list, we’ve got two or three players making big bets, big investments, and then probably three or four put a little bit in; not big but to have some exposure. And then there’s probably a number who are just supporting.
Respondent 2: Yes, that’s right. I think that’s become increasingly important. It’s near impossible now to raise funds unless you have a cornerstone investor who are willing to back it in size. (Brokers H and I)

Finding financing for life science startups is not only a long and drawn-out process, but the search for investment also reflects the fact that valuation itself is a collective and reflexive process. Stories generate expectations about startups that are actively and reflexively constituted by their investors, their advisors, and others, rather than simply being an effect of a business plan (Elder-Vass, 2021).

Financial analysts play an important role here too, constructing formal stories of firms, their markets, and valuation. In his ethnography of working as a financial analyst in an international bank, Leins (2018) emphasizes that one of the ‘most important skills in financial analysis’ is precisely ‘the ability to construct a coherent narrative’ (p. 64). In part, he argues, this relates to the fact that analysts are aware of their impact on political-economic decisions and choices – buying or selling shares, for example – which then problematizes the notion of fundamental value. Consequently, then, analysts have to be highly reflexive, looking ‘for information they think other market participants also will perceive as relevant’ (Leins, 2018, p. 82). Analysts dealing with technoscientific sectors play a similar role, as this informant, an analyst at a brokerage, highlighted:

So typically we’d write bigger kind of research notes on companies that we’re looking to IPO. If it’s obviously a medical firm we put out 50 page notes. And I mean that is sort of very typical just to highlight a very broad understanding of what is a new company. (Broker G)

The informant went on to state:

On the back of that I would be going out and meeting with investors to warm up the story and about four weeks later the company would actually go round. So we try and identify a group of investors that we think would be interested and we target the company to those. The investors come back and say ‘yeah, we quite like this, we want to put in this much money or have this much of the company’, and it’s our job to essentially build the book because quite often these companies want to raise money. (Broker G, 2012)

This informant is not only actively ‘warming up’ the story about the investee firm, but also generating investors (i.e. audience) who would be interested (Elder-Vass, 2021). Stories are an essential component in this active pitching process. And such stories are developed and honed over time so that, as the same informant explained, ‘by the time they go out to meet the investors, we’ve helped them refine their presentation’.

### Reflexive expectations and the financing of ‘exits’

The venture financing process ends with divestment, which is how venture funds make a return on their investment – usually through a trade sale (mostly) or IPO (rarely; Birch, 2017b). Valuation matters here, and Elder-Vass (2021) provides a useful outline of VC’s specificity with his distinction between ‘absolute’ valuation approaches (e.g. net present value) and ‘relative’ valuation approaches (e.g. market comparisons): Venture funders generally prefer the latter because it can lead to higher valuations at exit. Returns on venture investments tend to get split between LPs and GPs on an 80/20 basis; so, on top of their 2 percent annual management fee, GPs receive 20 percent of investment gains – called ‘carried interest’ (Cooiman, 2021; Shapin, 2008). However, this only happens after investments have returned 100 percent of the fund commitment. Venture investors have a tendency to make later-stage investments precisely because these investments are closer to exit – and therefore less risky with quicker returns. Generally, venture investors have very high expectations of returns on their investments, somewhere between 2.2 and 3.7 times the size of their investment over a three- to five-year period (Klonowski, 2018, p. 201), which militates against long-term thinking.

How expected ‘exit’ valuations are made and realized is an important issue; these expectations have to be sustained by startups through their responses to a number of funding rounds that stipulate particular milestone achievements (Hopkins, 2012). Consequently, startups are under pressure to identify an exit strategy early on with this mode of valuation, whether or not it is beneficial for the startup (Fabozzi, 2016). Moreover, exit strategies are increasingly configured by the expectations of higher achievable returns through trade sales rather than IPOs, which also configures the business plan and model at an early stage (Birch 2017b).

Constructing a story, honing it, pitching it, and creating an audience for it tells us something about VC as a mode of valuation: that startups are configured by a narrative framing that is both future-oriented and reflexive, understanding that futures are constructs of decisions, discourses, and practices in light of present fictional expectations (Beckert, 2016). However, the analytic focus on how the future is enacted in the present, which is evident in much of the expectations and futures literature (e.g. Beckert, 2013; Borup et al., 2006; Brown & Michael, 2003), does not address the important issue that the future is not only enacted in the present, but that bringing the future ‘into being’ necessitates a durational analysis that unpacks how temporal expectations are reflexively bound to particular practices and processes running temporally from the future to the present (Tellmann, 2020). As one informant commented, ‘you’ve got to start with what you want at the end and build the foundation back to it’ (ex-VC Investor N). Tellmann (2020, p. 347) argues that this entails material and organizational practices and processes extending between now and then, not binary relationship between two points in time (also Tutton, 2017). This argument reflects wider concerns with the process of ‘becoming an asset’ (Birch & Muniesa, 2020; Muniesa et al., 2017).

To analyse this further, I draw on an interview transcript sections that I think is particularly relevant for understanding the importance of reflexive expectations within a durational temporality. The informant responded to my question about the processes of valuation:

Yeah, for me it’s pretty simple. Not everyone would agree. But we start with the thesis that if we’re successful, what’s this company worth as an asset? So, you look at the market. The market sizing. The expected reimbursement, right? What an acquisitor will pay, all those kinds of things. And you say, you know, this company in four years, if we do all the things that we need to do to drive it to value, and typically for us a value inflection point is the end of phase two. Or for a medtech company, it’s … we’re in the market and we have somewhere between [C$] 10 and 20 million in revenues. So we use that as our benchmark, and we say, okay, if our company’s in that criteria zone, has had good phase two data, has had a good commercial launch and a good commercial strategy, what’s it worth? (Venture Investor S)

The informants start with the endpoint – much of which rests on a reflexive assessment of what the VC investor expects to get in a trade sale (Birch, 2017b) – and this comes to frame how they pursue their investment (also ex-VC Investor N). The informant specifically stated that ‘we’ve always planned our exits around trade sales, acquisitions’. Here, the expectations unfold over time (Tellmann, 2020), rather than at binary points. The informant went on to say:

Then we look at … so picking on [Firm X] for argument’s sake, $400 million [what it’ll be worth]. So now I’ll say, how much money am I going to have to deploy through the investment cycle to get to that inflection point that I now think and described as the value inflection point? So in … this is probably available data, but in most therapeutic companies, that number is about [C$] 67 million. In most medtech companies it’s about [C$] 55 million. So, when you do the math, in order to get kind of a risk adjusted reasonable rate of return, and assuming you leave something for management, founders and other people like that, you probably have to cycle back and say, how do I get a four or five X? Well, it means evaluation today can’t be much more than [C$ million] 40, right? Or 30 or whatever. Because do the math. Someone comes in and says 30, and I have to put in 50, now I’ve got a post money of 80, and I sell it for 400, that’s a five X. So that’s how we think about it. You know, how many … and so if I have to go through that cycle, and I start at 10, then I put in 20 for a post of 30, right? And then I assume that they’ve created value and I can get my evaluation up to 50, and afterwards another 30 for a post of 80, and I want my five X, 400 million, right? So. That’s how we look at it’.

The informant sets an amount – C$400 million – as their goal, reflecting the ‘value inflection point’ (i.e. exit) as what they would expect to get for a comparable firm in a comparable market at that particular point in the financing process. These comparables differ depending on the sector; in particular, medtech is lower because of the lower expected returns on capital than therapeutic startups, therefore requiring a higher multiple (Deloitte, 2019). That determines *how* they will invest – not just *what* sectors they will invest in – over the duration of their involvement. The informant then specifically turned to more quantitative valuation tools, like discounted cash flows:

It’s got nothing to do with the company talking about their DCFs and they’re discounted cash flows and all such bullshit. It means nothing. Like, it means nothing to me. What we care about is what do we think the exit value is. How many opportunities we’re going to have to exit at that value. When is that likely to happen? And how do we have to fund to that value inflection point? And again, we have a targeted rate of return. We want to make between four and six, you know, times our money on everything we do, right? The earlier the stage the higher the risk. We want to have the opportunity to make it eight or a 10. But we don’t do anything unless we see a four. Because we have a portfolio and we know within life sciences, biology’s biology and companies are going to fail, irrespective of strategy, irrespective of how good the management team is. Biology is biology. So we know a couple of our companies are going to fail in every portfolio based on biology, and then we know a couple of our companies are going to fail based on a lack of commercial execution, right?

The informant is particularly derisive about certain valuation devices (Doganova & Muniesa, 2015), focusing instead on funding to their ‘exit value’ as the key investment metric. While an exit is, by definition, a future event, the informant’s decisions reflect a durational approach (e.g. multiple investments at different stages) in their effort to reach a particular inflection point (Tellmann, 2020). They finish by saying:

And so when we think about our pricing model, we have to make sure that we take into account a certain number of failures, and we can still generate a reasonable rate of return for our limited partners. So, yeah, we do DCFs and we look at cash flows. But that’s not really how we’re thinking about it. Right? We’re thinking about what’s the X multiple likely to be. Is that multiple validated in the market by comparable transactions? Do we think there’s enough potential bidders to make it a process that will drive that number? I mean we’ll never do a deal where we think there’s only one acquisitor. Just not interested. And we’ll never do a deal where we think the only exit is to a public market. (Venture Investor S)

As the long interview quote above illustrates, reaching an ‘exit’ is a key point in the VC financing process, achieved after a series of ongoing decisions and actions that configure valuation over a duration (Tellmann, 2020). Scholars such as Andersson et al. (2010) describe this as a relay as one funder passes the ‘baton’ onto another until exit (also Birch, 2017b; Styhre, 2021). Here, thinking of VC as a mode of valuation entails going beyond a notion that future expectations are primarily a response to the inherent (or perceived) uncertainties in technological change or capitalism. As Muniesa and Doganova (2020, p. 100) point out, this analytical centering of the future in the present ends up cementing a notion of fundamental value derived from calculative practices and devices like discounting (also Doganova, 2018; Muniesa et al., 2017); it reinforces the very financial thinking that it is meant to challenge. Muniesa and Doganova (2020) go on to argue that when valuation is theorized as an anticipation of future yield it justifies and legitimates the value assigned to investment, in particular, and does not do enough to unpack how ‘the craft of monetary value-making is responsible for producing a certain idea of time’ (p. 108). Consequently, it is worthwhile to consider *how* VC financiers who make investments understand, reflexively, the ‘exit’ point in this process.

An ‘exit’ is, then, a future expectation, being described as an ‘inflection point’ by informants and others; more specifically, it is a future (or endpoint) to which VC reflexively orient their activities, decisions, and practices. This can lead to a disjuncture in the stories told about startups, moving from a highly positive narrative towards one that is more pessimistic (Chapple et al., 2021):

At the beginning it’s great, you know, you can raise money with a great story and a great patent portfolio and, you know, saying you’re going to revolutionize this area of medicine or that area of medicine. But the long haul is always very, very hard. So my end view is that you’re better off being venture funded, and try to get your technology into the hands of larger pharma as a licensing transaction as soon as you reasonably can get a return which is going to justify the investments made to date. (VC Investor K)

This informant was a VC investor in a generalist technology fund who invested in life science firms, primarily in the UK, alongside other technology sectors. They exemplified the reflexive focus of VC investors on finding the ‘cleanest’ exit, aiming for a ‘single transaction’ that would reduce tax implications through returns from capital gains rather than an ongoing ‘succession of license payments paid out as dividends’. Another informant demonstrated similar reflexive concern with taxation with a blunt statement that: ‘you want to structure your company, your organization, appropriately up front in order to ensure that the IP is in the right jurisdiction in order to pay as little tax as possible’ (Accountant O).

A trade sale is an easier story to manage in this regard, making it easier to enrol other social actors in the narrative (Kenney & Zysman, 2019), while ensuring a higher return on investment because of the ‘knowledge premium’. As Birch (2017b) explains, the knowledge premium represents the difference between the technoscientific knowledge of specialists (e.g. other hi-tech firms), who will know something about the firm they are acquiring, versus the knowledge of generalists (e.g. institutional investors), who might know next to nothing. Here, reflexive expectations are critical for valuation because VC investors can act upon their understanding of a particular startup’s story by framing and reframing it for suitable acquirers. This is vital, as an informant noted, because:

that’s the problem we face, that’s a problem lots of people face … that’s one of the reasons that biotech companies go bust. They’re easy to start, they all tell fantastic stories which are attractive to investors, but they’re hard to pump all the way to an exit point so there is a lot of attrition. (VCT Investor E)

## Conclusion

The venture capital industry is an important topic for science and technology studies because it is becoming a key driver of technoscientific innovation, and the dynamics that underpin VC as a mode of valuation produce problematic effects. My aim in this article was to analyse this VC mode of valuation by examining the reflexivity of social actors in the VC financing process. My focus was on analysing how these actors understand, use, and deploy stories in VC financing in order to analyse their reflexive framing of future expectations in investment decisions. As such, I sought to build on a growing literature in science and technology studies, economic sociology, and organization studies that examines the constitutive and performative nature of future expectations as they generate forms of collective meaning and legitimacy in order to resolve the inherent uncertainties in hi-tech sectors. This analytical attention on the future and future expectations goes some way to explain what and why stories matter in the venture capital process, but it does not get at how actors themselves understand and act upon those stories.

Throughout the VC financing process, stories and narratives are deployed at different points and with different objectives. Actors are deliberately and consciously generating stories or acting reflexively in relation to them as part of their investment expectations, decisions, and strategies. These ‘reflexive expectations’, as I theorize them, are different from the more performative notion of expectations in the existing literature. Reflexive expectations start with ‘good stories’ about the *promises* inherent in certain hi-tech sectors, specifically in the life sciences – which is generically characterized as offering ‘a cure for cancer’. As the financing process unfolds in the life sciences sector, actors construct new stories or amend old ones as a way to make valuation judgements and to attract investment. Here, valuation and investment are both part of a collective and reflexive process entailing the creation of an audience, or ‘asset circle’ (Elder-Vass, 2021), through the development of prospectuses, roadshows, meetings, analyst reports, and so on (i.e. story materials). Finally, as a mode of valuation VC entails a set of durational practices (Tellmann, 2020) that investors use to exit their investments. Overall, this financing process illustrates how expectations are reflexively materialized *along* a timeframe that is durational, rather than binary (i.e. future-present). From this perspective, the stories that actors create, tell, retell, and contest are ways to materialize future expectations in durational terms, as much as they are ways to deal with uncertainty (Tellmann, 2020; Loza, 2021). And they know that stories are how they can materialize temporal expectations, which they do throughout the duration of the financing process.

Despite the attention on futures, then, the literature on expectations needs to expand its analytical lens; more specifically, there is a need for further examination of the reflexivity of social actors who both have future expectations *and* knowingly deploy expectations as a way to configure *and* reconfigure the temporalities they encounter in durational processes (Tellmann, 2020). There remains a need to analyse how expectations are reflexively performed but also contested and reworked throughout the financial cycle (see Chapple et al., 2021). Stories play a key role in opening up new opportunities – or closing down options – through their transformation of valuation and investment processes. Similarly, Muniesa and Doganova (2020) argue that financial valuation legitimates certain metrics (e.g. future yield) that then produces a particular temporality, which reinforces the original financialized perception of the world. This, necessarily, has problematic implications; for example, there is an increasing disconnect between the technological expectations and financial expectations, where the latter come to matter more – socially, politically, culturally – than the former.

## References

[bibr1-03063127221118372] Andersson T. Gleadle P. Haslam C. Tsitsianis N. (2010). Bio-pharma: A financialized business model. Critical Perspectives in Accounting, 21(7), 631–641.

[bibr2-03063127221118372] Balzam G. Yuran N. (2022). Assetization and the logic of venture capital, or why Facebook does not ‘feel’ like a monopoly to Zuckerberg. Science as Culture, 31(1), 107–120.

[bibr3-03063127221118372] Beckert J. (2011). The transcending power of goods: Imaginative value in the economy. In Beckert J. Aspers P. (Eds.), The worth of goods (pp. 106–128). Oxford University Press.

[bibr4-03063127221118372] Beckert J. (2013). Imagined futures: Fictional expectations in the economy. Theory & Society, 42(3), 219–240.

[bibr5-03063127221118372] Beckert J. (2016). Imagined futures: Fictional expectations and capitalist dynamics. Harvard University Press.

[bibr6-03063127221118372] Beckert J. (2020). The firm as an engine of imagination: Organizational prospection and the making of economic futures. Organizational Theory, 2(2), 1–21.

[bibr7-03063127221118372] Birch K. (2016). Innovation, regional development and the life sciences: Beyond clusters. Routledge.

[bibr8-03063127221118372] Birch K. (2017a). Introduction: Techno-economic assumptions. Science as Culture 26(4), 433–444.

[bibr9-03063127221118372] Birch K. (2017b). Rethinking value in the bio-economy: Finance, assetization and the management of value. Science, Technology and Human Values, 42(3), 460–490. 10.1177/0162243916661633 PMC5390941 28458406

[bibr10-03063127221118372] Birch K. Muniesa F. (Eds.). (2020). Assetization: Turning things into assets in technoscientific capitalism. MIT Press.

[bibr11-03063127221118372] Borup M. Brown N. Konrad K. van Lente H. (2006). The sociology of expectations in science and technology. Technology Analysis and Strategic Management, 18(3/4), 285–298.

[bibr12-03063127221118372] Brown N. Michael M. (2003). A sociology of expectations: Retrospecting prospects and prospecting retrospects. Technology Analysis and Strategic Management, 15(1), 3–18.

[bibr13-03063127221118372] Brown N. Rappert B. Webster A. (Eds.). (2000). Contested futures: A sociology of prospective technoscience. Ashgate.

[bibr14-03063127221118372] Canadian Venture Capital Association. (2021). Canadian VC & PE market overview. Retrieved May 2021, from https://www.cvca.ca/research-insight/market-reports

[bibr15-03063127221118372] CB Insights. (2021) The state of venture: Global 2021. Author.

[bibr16-03063127221118372] Chapple D. Pollock N. D’Adderio L. (2021). From pitching to briefing: Extending entrepreneurial storytelling to new audiences. Organization Studies, 43(5), 773–795.

[bibr17-03063127221118372] Cooiman F. (2021). Veni vidi VC: The backend of the digital economy and its political making. Review of International Political Economy. Advance online publication. 10.1080/09692290.2021.1972433

[bibr18-03063127221118372] Czarniawska B. (2004). Narratives in social science research. SAGE.

[bibr19-03063127221118372] Deloitte. (2019). Return on capital performance in life sciences and health care. Author.

[bibr20-03063127221118372] Doganova L. (2018). Discounting and the making of the future. In Beckert J. Bronk R. (Eds.), Uncertain futures (pp. 278–297). Oxford University Press.

[bibr21-03063127221118372] Doganova L. Eyquem-Renault M. (2009). What do business models do? Innovation devices in technology entrepreneurship. Research Policy, 38(10), 1559–1570.

[bibr22-03063127221118372] Doganova L. Muniesa F. (2015). Capitalization devices: Business models and the renewal of markets. In Kornberger M. Jutesen L. Mouritsen J. Madsen A. K. (Eds.) Making things valuable (pp. 109–125). Oxford University Press.

[bibr23-03063127221118372] Elder-Vass D. (2021). Assets need audiences: How venture capitalists boost valuations by recruiting investors to asset circles. Finance & Society, 7(1), 1–19.

[bibr24-03063127221118372] Fabozzi F. (2016). Entrepreneurial finance and accounting for high-tech companies. MIT Press.

[bibr25-03063127221118372] Fortun M. (2001). Mediated speculations in the genomics futures markets. New Genetics and Society, 20(2), 139–156.

[bibr26-03063127221118372] Fortun M. (2008). Promising genomics: Iceland and deCODE genetics in a world of speculation. University of California Press.

[bibr27-03063127221118372] Geiger S. (2020). Silicon Valley, disruption, and the end of uncertainty. Journal of Cultural Economy, 13(2), 169–184.

[bibr28-03063127221118372] Giddens A. (1984). The constitution of society. University of California Press.

[bibr29-03063127221118372] Hedgecoe A. Martin P. (2003). The drugs don’t work: Expectations and the shaping of pharmacogenetics. Social Studies of Science, 33(3), 327–364. 14621671 10.1177/03063127030333002

[bibr30-03063127221118372] Hellman J. (2022). Big tech’s ‘voracious appetite’ or entrepreneurs who dream of acquisition? Regulation and the interpenetration of corporate scales. Science as Culture, 31(1), 149–161.

[bibr31-03063127221118372] Hogarth S. (2017). Valley of the unicorns: Consumer genomics, venture capital and digital disruption. New Genetics and Society, 36(3), 250–272.

[bibr32-03063127221118372] Hopkins M. (2012). Exploring finding routes for therapeutics firms. In O’Neill M. Hopkins M. (Eds.), A biotech manager’s handbook (pp. 191–255). Woodhead.

[bibr33-03063127221118372] Kenney M. Zysman J. (2019). Unicorns, Cheshire cats, and the new dilemmas of entrepreneurial finance. Venture Capital, 21(1), 35–50.

[bibr34-03063127221118372] Klonowski D. (2018). The venture capital deformation. Palgrave Macmillan.

[bibr35-03063127221118372] Leins S. (2018). Stories of capitalism. University of Chicago Press.

[bibr36-03063127221118372] Liu W. (2020). Abolish Silicon Valley. Repeater Books.

[bibr37-03063127221118372] Loza O. (2021). Constructing a ‘just’ future: Technoutopian visions of the graduate labour market [Conference session]. Nordic Science and Technology Studies Conference, 20-21 May 2021, Copenhagen.

[bibr38-03063127221118372] Mallaby S. (2022). The power law: Venture capital and the art of disruption. Penguin.

[bibr39-03063127221118372] Mirowski P. (2012). The modern commercialization of science as a passel of Ponzi schemes. Social Epistemology, 26(3–4), 285–310.

[bibr40-03063127221118372] Muniesa F. Doganova L. (2020). The time that money requires: Use of the future and critique of the present in financial valuation. Finance & Society, 6(2), 95–113.

[bibr41-03063127221118372] Muniesa F. Doganova L. Ortiz H. Pina-Stranger A. Paterson F. Bourgoin A. Ehrenstein V. Juven P.-A. Pontille D. Saraç-Lesavre B. Yon G. (2017). Capitalization: A cultural guide. Presses des Mines.

[bibr42-03063127221118372] National Venture Capital Association. (2021). 2021 yearbook. Author. https://nvca.org/wp-content/uploads/2021/03/NVCA-2021-Yearbook.pdf

[bibr43-03063127221118372] Owen G. Hopkins M. (2016). Science, the state, and the city. Oxford University Press.

[bibr44-03063127221118372] Palo T. Mason K. Roscoe P. (2020). Performing a myth to make a market: The construction of the ‘magical world’ of Santa. Organization Studies, 41(1), 53–75.

[bibr45-03063127221118372] Pisano G. (2006). Science business: The promise, the reality, and the future of biotech. Harvard Business School Press.

[bibr46-03063127221118372] Pfotenhauer S. Laurent B. Papageorgiou K. Stilgoe J. (2022). The politics of scaling. Social Studies of Science, 52(1), 3–34. 34625011 10.1177/03063127211048945 PMC8771885

[bibr47-03063127221118372] Sauter M. (2020). A businessman’s risk: The construction of venture capital at the center of US high technology [PhD Thesis]. McGill University.

[bibr48-03063127221118372] Selin C. (2008). Sociology of the future: Tracing stories of technology and time. Sociology Compass, 2(6), 1878–1895.

[bibr49-03063127221118372] Shapin S. (2008). The scientific life. University of Chicago Press.

[bibr50-03063127221118372] Styhre A. (2015). Financing life science innovation. Palgrave Macmillan.

[bibr51-03063127221118372] Styhre A. (2021). The financialized economy. Routledge.

[bibr52-03063127221118372] Tellmann U. (2020). Beyond performativity: Material futures of finance. Economy and Society, 49(3), 345–363.

[bibr53-03063127221118372] Tellmann U. (2022). The politics of assetization: From devices of calculation to devices of obligation. Distinktion, 23(1), 33–54.

[bibr54-03063127221118372] Tutton R. (2017). Wicked futures: Meaning, matter and the sociology of the future. The Sociological Review, 65(3), 478–492.

[bibr55-03063127221118372] Van Lente H. (1993). Promising technology: The dynamics of expectations in technological developments. Eburon.

[bibr56-03063127221118372] Van Lente H. Rip A. (1998). The rise of membrane technology: From rhetorics to social reality. Social Studies of Science, 28(2), 221–254.

[bibr57-03063127221118372] Yin E. (2021). Today’s tweet thread is about how LPs (fund investors) make money … [Twitter thread]. Twitter. https://threadreaderapp.com/thread/1380253396880351232.html

